# Developing an optimized method for biofilm extraction from microplastic surfaces for high-efficiency analysis of adherent bacterial communities

**DOI:** 10.1128/aem.00416-26

**Published:** 2026-04-29

**Authors:** Hieu Hoai Vo, Thao Thanh Le, Tu Van Nguyen, Jennifer Scott, Tony Gutierrez, Michel J. Kaiser, Huong Thi Thuy Ngo

**Affiliations:** 1 Environmental Chemistry and Ecotoxicology Lab, Phenikaa School of Engineering, Phenikaa University, Ha Noi, Vietnam; 2 Yersin University of Da Lat, Lam Dong, Vietnam; 3 National Institute of Hygiene and Epidemiology 310750, Ha Noi, Vietnam; 4 Faculty of Biotechnology, Chemistry and Environmental Engineering, Phenikaa School of Engineering, Phenikaa University, Ha Noi, Vietnam; 5 Institute of Mechanical, Process and Energy Engineering, School of Engineering and Physical Science, Heriot-Watt University, Institute of Mechanical, Edinburgh, United Kingdom; 6 The Lyell Centre, School of Energy, Geoscience, Infrastructure and Society, Heriot-Watt University, Edinburgh, United Kingdom; University of Minnesota Twin Cities, St. Paul, Minnesota, USA

**Keywords:** microplastics, biofilm extraction, plastisphere, pathogens, antibiotic resistance, aquatic ecosystems

## Abstract

Microplastics (MiPs, ×5 mm in size) harbor complex biofilms that facilitate pathogen dissemination, yet standardized extraction protocols are lacking. Here, we developed and optimized a method for biofilm extraction from environmentally weathered MiPs. To reflect real-world conditions, the protocol was applied directly to bulk, heterogeneous, field-collected MiP mixtures (size range: 80 µm–5 mm) without prior sorting by polymer type or morphology. By optimizing extraction buffers, mechanical disruption, and MiP quantities (100–150 particles), we established an optimal protocol combining phosphate-buffered saline with 0.1% Tween 80, ultrasonication (40 kHz, 10 min), vortexing with glass beads, and a two-cycle extraction-disaggregation workflow. This approach involves an initial extraction followed by a repeated, exhaustive extraction step designed to maximize the recovery of recalcitrant biofilm residues. This protocol markedly enhanced recovery of viable, culturable cells, delivering a 2,950-fold enhancement in the recovery of viable, culturable cells (evaluated via CFU counts; 28,020 ± 11,034 CFU MiP⁻¹) vs. conventional PBS extraction (9.5 ± 3 CFU MiP⁻¹) and 102-fold vs. passive extraction (274 ± 59 CFU MiP⁻¹). The 10-min sonication empirically maximized viable cell recovery within the tested duration range. The two-step protocol with Tween 80-mediated disaggregation proved critical, increasing recovery 208-fold by disaggregating biofilm fragments. While DNA yields (26.5 ± 3.93 ng µL⁻¹) were sufficient for targeted PCR-based pathogen detection (*Aeromonas* spp., *Salmonella enterica*), the co-extraction of complex environmental matrices (*A*_260_/*A*_280_ ratio: 0.17–0.19) strictly requires an additional purification step prior to next-generation sequencing. Validation across contrasting aquatic environments confirmed the method’s robustness. Comparative analysis demonstrates that conventional single-step approaches fail to recover the majority of viable cells trapped within weathered MiP biofilms. This optimized and validated protocol provides a critical methodological foundation for investigating plastisphere microbial ecology and pathogen transport dynamics, supporting evidence-based risk assessment of MiP contamination, especially public health risks associated with microplastic pollution.

**IMPORTANCE:**

Microplastic-associated biofilms (the “plastisphere”) serve as vectors for waterborne pathogens and antibiotic resistance genes; however, the persistent use of inadequate extraction methods has systematically underestimated microbial abundance, presenting a critical barrier to global environmental risk assessment. By overcoming the limitations of conventional extractions—which fail to penetrate recalcitrant extracellular polymeric matrices on environmentally weathered microplastics—our standardized methodology liberates previously undetectable bacterial populations. The ability to accurately quantify these hidden communities, including key pathogens like *Aeromonas* spp. and *Salmonella enterica*, fundamentally transforms our understanding of microplastics as hidden biological reservoirs. Ultimately, this methodological advancement bridges a critical gap in microbial ecology, delivering the reliable, quantitative data strictly required by policymakers, environmental agencies, and public health officials to establish evidence-based guidelines mitigating the impacts of microplastic pollution on global water systems.

## INTRODUCTION

Microplastics (MiPs) are now recognized as pervasive contaminants in aquatic environments and can serve as surfaces for microbial colonization (1, 2). In aquatic systems, MiPs rapidly develop biofilms comprised of complex and dynamic microbial consortia embedded within self-secreted extracellular polymeric substances (EPS) (3–5). These biofilms, collectively termed the “plastisphere” since the foundational work by Zettler (6), differ markedly from microbial assemblages on natural particles or in the surrounding water column, forming novel ecological niches that influence local biogeochemical cycles (7). Understanding the formation, composition, and ecological function of MiP-associated biofilms has become increasingly important because they may mediate contaminant transport (8) and biodegradation (9), pathogen dissemination (10, 11), and gene exchange processes (12) in aquatic systems.

Growing evidence indicates that MiPs act as vectors for pathogenic and antibiotic-resistant bacteria, posing potential threats to aquatic organisms and human health under the One Health paradigm (13). MiPs can provide stable, nutrient-enriched microhabitats that facilitate the attachment, survival, and proliferation of opportunistic pathogens, including *Pseudomonas*, *Escherichia*, *Acinetobacter*, and *Vibrio* species (14, 15). Because of their clinical relevance, these specific taxa, alongside *Aeromonas* and *Salmonella*, are frequently utilized as critical “indicator species” to evaluate plastisphere-associated health risks. Equally concerning, plastisphere communities are often enriched with antibiotic resistance genes (ARGs) encoding resistance to multiple drug classes (14, 16). The co-occurrence of ARGs and pathogens on MiPs suggests these particles may serve as hotspots for horizontal gene transfer and reservoirs for resistance dissemination across ecosystems (17, 18). Consequently, reliable characterization of MiP-associated microbial communities is essential for assessing ecological and public health risks (1).

Biofilm formation on MiPs is influenced by polymer physicochemical properties (type, size, and surface roughness), water chemistry, and environmental conditions (19). Plastic polymers generally support more diverse and complex biofilm communities than natural materials (20, 21), with polymer-specific characteristics affecting microbial attachment, succession, and metabolic activity (2).

The study of plastisphere communities requires cultivation-dependent and/or cultivation-independent approaches (22). However, a key bottleneck that remains at the very start of these investigations is obtaining the efficient extraction of biofilms from MiP surfaces without compromising cell viability or DNA integrity. In natural settings, MiPs are typically weathered and form complex surface layers (4, 20). The physical nature of the EPS matrix on these weathered polymers presents a formidable mechanistic challenge for extraction. Commonly used methods—including basic sonication (23, 24), vortexing (25), shaking (26), and methods using glass beads (27) or surfactants such as Tween 80 (28)—often fail because the robust EPS acts as an adhesive shield. Low-energy physical treatments result in incomplete detachment, leaving substantial numbers of cells trapped within the EPS matrix on microcracked surfaces. Conversely, applying excessive mechanical stress to force complete detachment can damage cells or shear DNA (29, 30), consequently biasing downstream microbial analyses. Compounding this bottleneck further, these procedures rarely report quantitative metrics such as biofilm extraction efficiency, viable cell recovery, or DNA yield. The lack of methodological standardization severely hampers reproducibility and cross-study comparison.

Recent efforts have attempted to improve biofilm extraction methods. Building upon the foundational work of Debeljak et al. (22) and Stevenson et al. (31), who utilized environmentally weathered polymers to investigate extraction methods, our study advances the field by systematically optimizing each variable of the biofilm extraction workflow. However, their work was fundamentally comparative in design—testing fixed parameters across different approaches—rather than systematically optimizing individual variables to maximize performance. While Stevenson et al. effectively utilized environmentally weathered polymers (PE, PS, and PP), their study was fundamentally comparative—evaluating fixed parameters across different approaches—rather than systematically optimizing the individual variables of the physical detachment process (31). Furthermore, comprehensive quantitative performance metrics (such as absolute extraction efficiency or viable cell recovery rates per particle) were not extensively reported, which limits the standardization and reproducibility of the protocol.

Similarly, Debeljak evaluated DNA extraction efficiency across commercial kits and observed that DNA recovery scaled with particle surface area (22). However, their study focused on downstream chemical lysis optimization rather than upstream physical biofilm detachment—a critical distinction. Their simple physical extraction (shaking in PBS) likely left substantial biofilm material on particle surfaces, particularly for weathered MiPs with rough, microcracked surfaces that trap cells. Moreover, neither Stevenson nor Debeljak addressed the dual optimization required for both culture-dependent pathogen isolation (requiring viable cell recovery) and molecular analyses, a critical gap for comprehensive plastisphere characterization under One Health frameworks, where accurate, unbiased data are essential prerequisites for predictive risk modeling (13, 18).

At present, more than a decade after the plastisphere was first characterized, there is no standardized, validated method that (i) systematically optimizes each critical parameter of the biofilm extraction process, (ii) achieves high biofilm removal efficiency while preserving microbial viability, (iii) performs consistently across diverse environmental conditions (freshwater vs. sediment, aerobic vs. anaerobic), and (iv) enables both culture-based and molecular characterization from a single extraction. This persistent methodological gap systematically underestimates microbial abundance on MiPs, thereby constraining the development of accurate One Health risk assessment models regarding pathogen carriage and antimicrobial resistance. Here, we developed and rigorously validated an optimized biofilm extraction protocol that systematically addresses these limitations. By optimizing mechanical disruption and proposing a two-cycle extraction-disaggregation workflow designed to maximize the recovery of recalcitrant biofilm residues, this method achieves substantially enhanced extraction efficiency while maintaining dual functionality for microbiological and molecular applications.

## RESULTS

### Effect of the extraction solution on biofilm extraction efficiency

**Fig 1:**
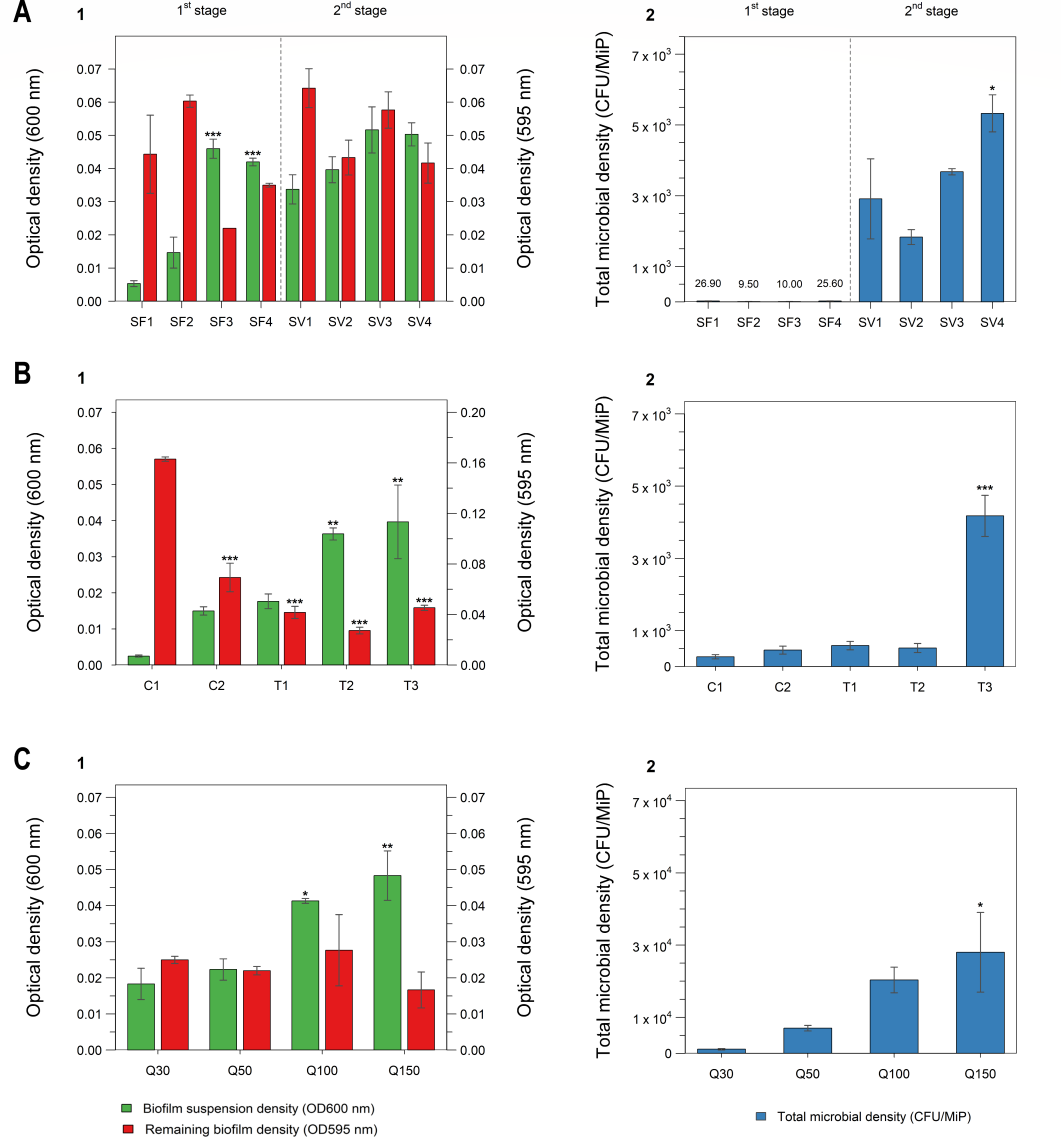
Optimization of biofilm extraction-disaggreation from microplastic surfaces. (A1, B1, and C1) Extraction efficiency assessed by optical density of the extracted biofilm suspension (OD_600_; green bars) and residual crystal-violet-stained biofilm remaining on microplastics (OD_595_; red bars) under varying conditions: (**A**) extraction solution formulations (SF1–SF4, SV1–SV4), (**B**) ultrasonication parameters (C1–C2, T1–T3), and (**C**) microplastic particle loadings (Q30–Q150). (A2, B2, and C2) Corresponding viable microbial counts (CFU MiP^−1^) obtained from microplastic surfaces for each experimental condition. Significant differences in extraction efficiency (A1, B1, and C1) and microbial counts (A2, B2, and C2) relative to the lowest observed value are indicated (mean ± SEM, *n* = 3; *P < 0.05, **P < 0.01, ***P < 0.001).

Four extraction solutions were systematically evaluated during the initial detachment step: 0.85% (wt/vol) NaCl (SF1), 1× PBS (SF2), 1× PBS + 0.1% (vol/vol) Tween 20 (SF3), and 1× PBS + 0.1% (vol/vol) Tween 80 (SF4), each coupled with mechanical disruption (ultrasonication + vortexing with glass beads). Biofilm extraction efficiency (Fig. 1) was quantified using two complementary metrics: optical density of the biofilm suspension (OD_600_), reflecting detached biomass, and residual biofilm measured by crystal violet staining (OD_595_).

Surfactant-containing solutions (SF3 and SF4) achieved superior biofilm removal (OD_600_ = 0.046 ± 0.003 and 0.042 ± 0.001; OD_595_ = 0.022 ± 0.00 and 0.035 ± 0.001, respectively) compared to SF1 (one-way ANOVA for OD_600_: *F*(3, 8) = 49.97, *P* < 0.001; for OD_595_: *F*(3, 8) = 7.30, *P* = 0.011; Tukey’s HSD: *P* < 0.05 for both SF3 and SF4 vs. SF1) (Fig. 1A (1–2)). However, viable cell recovery (CFU MiP^−1^) showed unexpected patterns: saline (SF1, 26.9 ± 7.4 CFU MiP^−1^) and SF4 (25.6 ± 8.2 CFU MiP^−1^) outperformed SF3 (PBS + Tween 20: 10.0 ± 3.2 CFU MiP^−1^) and PBS alone (SF2, 9.5 ± 3.0 CFU MiP^−1^) (Fig. 1A2). Although SF1 achieved good recovery of viable cells, it proved inefficient for complete detachment of the biofilm (i.e., low biofilm detachment efficiency).

This critical discrepancy indicated that biofilm detachment and viable cell recovery are mechanistically distinct processes. Specifically, we hypothesized an initial disaggregation bottleneck, meaning that while surfactant-mediated mechanical disruption effectively detached the EPS matrix from the plastic (yielding high OD_600_), the viable cells remained tightly trapped within these suspended multicellular aggregates, unable to form individual colonies. Furthermore, it is important to note early on that relying primarily on CFU counts inherently captures only the culturable fraction, excluding viable-but-non-culturable (VBNC) populations, and may selectively favor stress-tolerant taxa.

To address this disaggregation bottleneck, a second optimization step evaluated disaggregation buffer (PBS vs*.* PBS + 0.1% Tween 80) and mechanical sequence effects on the pooled, detached biofilm suspensions (SV1–SV4; Table 1). This approach yielded dramatic improvements, with viable recovery increasing to 1.8–5.3 × 10³ CFU MiP^−1^—representing a 208-fold increase over the use of SF4, and 561-fold increase over the use of PBS alone (SF2) in Step 1 (Fig. 1A2). Treatment SV4 (PBS + 0.1% Tween 80, sonication followed by vortex) achieved optimal performance (5.3 × 10³ CFU MiP^−1^, one-way ANOVA: *F*(3, 8) = 32.19, *P* < 0.001; Tukey’s HSD: *P* < 0.001 vs. SV1), demonstrating that surfactant-mediated disaggregation of biofilm was essential for maximizing viable cell recovery. Based on these findings, SF4 extraction followed by SV4 disaggregation treatment was selected for the optimized protocol.

**TABLE 1:** Experimental design for optimizing biofilm extraction from microplastics: comparison of extraction solutions and mechanical processing sequences across two sequential extraction-disaggregation^*a*^

Treatment	Extraction step	Extraction/dilution solution	Mechanical processing order
FS1	First	0.85% NaCl	Sonication → Vortex
FS2	First	1× PBS	Sonication → Vortex
FS3	First	1× PBS + 0.1% Tween 20	Sonication → Vortex
FS4	First	1× PBS + 0.1% Tween 80	Sonication → Vortex
SV1	Second	1× PBS	Vortex → Sonication
SV2	Second	1× PBS	Sonication → Vortex
SV3	Second	1× PBS + 0.1% Tween 80	Vortex → Sonication
SV4	Second	1× PBS + 0.1% Tween 80	Sonication → Vortex

^*a*^ Step 1 (detachment) evaluated four extraction solutions; Step 2 (disaggregation) compared processing order effects using selected solutions from Step 1. All treatments included sonication (40 kHz, 10 min) and vortexing (3,000 rpm, 2× 30s). Step 2 applied the fresh extraction solution to microplastics after Step 1 completion.

Having established the optimal extraction solution and disaggregation protocol, we next evaluated mechanical disruption parameters.

### Effect of sonication duration on extraction efficiency

To determine the optimal mechanical disruption intensity, five treatments were systematically compared: passive extraction (C1), vortexing only (C2, 2× 30 s), and combined ultrasonication (40 kHz, 100 W) with vortexing for 3 min (T1), 5 min (T2), or 10 min (T3) (Fig. 1C and D).

Passive extraction (C1) yielded minimal recovery (274 CFU MiP^−1^, residual OD_595_ = 0.163), while C2 showed moderate improvement (458 CFU MiP^−1^, residual OD_595_ = 0.07) but remained substantially less effective than combined sonication–vortexing treatments. Among sonication treatments, T3 (10 min) achieved maximum efficiency with 4,175 ± 570 CFU MiP^−1^—15.2-fold higher than C1, and 9.1-fold higher than C2 (Fig. 1B [1-2]). Shorter durations yielded intermediate values (T1: 585 CFU MiP^−1^; T2: 516 CFU MiP^−1^). T3 exhibited significantly higher recovery than both T1 and T2 (one-way ANOVA: *F*(4, 10) = 37.59, *P* < 0.001; Tukey’s HSD: *P* < 0.001), while T1 and T2 showed no significant difference (Tukey’s HSD: *P* = 1.0).

Optical density measurements confirmed this trend with T3 producing maximum suspended biomass (OD_600_ = 0.040 ± 0.005, one-way ANOVA: *F*(4, 10) = 10.73, *P* = 0.001; Tukey’s HSD: *P* < 0.01) with minimal surface residue (OD_595_ = 0.046 ± 0.002, one-way ANOVA: *F*(4, 10) = 90.66, *P* < 0.001; Tukey’s HSD: *P* < 0.001) (Fig. 1B [2]). Critically, sustained CFU viability at 10-min exposure demonstrates that 40 kHz ultrasonication at 100 W effectively disrupts biofilms through cavitation-induced shear forces while preserving cell integrity—a key requirement for culture-based pathogen isolation. Based on these results, 10-min ultrasonication was selected as optimal for MiP biofilm extraction.

With the extraction buffer and sonication parameters optimized, we determined the minimum MiP quantity required for reliable analyses.

### Effect of microplastic particle quantity on biofilm extraction

Four quantified sets of MiPs (30, 50, 100, and 150 particles per set, named as Q30, Q50, Q100, and Q150, respectively; *n* = 6 for each replicate) were evaluated using the optimized extraction protocol to establish minimum loading requirements for reliable downstream analyses. MiP quantity strongly influenced biofilm recovery (Fig. 1C). Using spectrophotometry to assess biofilm extraction, absorbance measurements (OD_600_) increased with increasing MiP particle batches—from 0.018 ± 0.004 (Q30) to 0.048 ± 0.007 (Q150), a significant difference (one-way ANOVA: *F*(3, 8) = 11.28, *P* < 0.01), with Q150 showing significantly higher absorbance than Q30 (Tukey’s HSD: *P* < 0.01) and Q50 (Tukey’s HSD: *P* < 0.05). This was matched also by a significant increase in viable cell counts by 24-fold with increasing particle batches (one-way ANOVA: *F*(3, 8) = 4.45, *P* < 0.05)—from 1,170 ± 205 CFU MiP^−1^ (Q30) to 28,020 ± 11,034 CFU MiP^−1^ (Q150) (Tukey’s HSD: *P* < 0.05).

Importantly, residual biofilm (OD_595_) attached to the MiPs remained uniformly low (0.017–0.028, one-way ANOVA: *F*(3, 8) = 0.715, *P* > 0.05), with no significant differences between any particle loading groups (Tukey’s HSD: all *P* > 0.05), confirming consistent extraction efficiency across all particle loadings. Relative to the conventional PBS extraction method (SF2), the optimized protocol markedly increased viable cell recovery, yielding a 123-fold improvement with 30 particles (Q30), 737-fold with 50 particles (Q50), 2,141-fold with 100 particles (Q100), and 2,950-fold with 150 particles (Q150). Increasing particle counts provides greater absolute biomass yield by increasing total available surface area, which enhances the probability of detecting low-abundance targets in downstream PCR assays. However, this reflects greater total input material rather than improved extraction mechanics per particle.

DNA yields increased proportionally with the number of MiP particles processed, rising from 13.9 ± 0.87 ng µL⁻¹ in Q30 to 26.5 ± 3.93 ng µL⁻¹ in Q150 (one-way ANOVA: *F*(3, 8) = 23.04, *P* < 0.001). DNA concentrations obtained from Q150 were significantly higher than those from Q30–Q50 (Tukey’s HSD: *P* < 0.05). DNA purity ratios (*A*_₂₆₀_/*A*_₂₈₀_ = 0.17–0.19, one-way ANOVA: *F*(3, 8) = 4.85, *P* < 0.05) were extremely low, reflecting massive co-extraction of proteinaceous EPS components and solvent contaminants inherent to environmental biofilm matrices. Despite these low purity ratios, the extracted raw DNA empirically supported successful targeted PCR amplification. While we screened the extracts for a panel of four ubiquitous gram-negative plastisphere indicators (*E. coli*, *P. aeruginosa*, *Aeromonas* spp., and *S. enterica*), we successfully detected specific 16S rRNA marker genes for *Aeromonas* spp. and *S. enterica* in these environmental samples (Fig. S1). However, we explicitly acknowledge that these raw DNA extracts are not of sufficient quality for sensitive, highly efficient molecular workflows (e.g., next-generation sequencing [NGS]) without rigorous downstream purification.

Based on these findings, 100–150 MiP particles per replicate are recommended for robust biofilm characterization (Table 2), providing sufficient biomass (>15,000 CFU MiP^−1^) and DNA yield (>20 ng µL⁻¹), along with acceptable reproducibility.

**TABLE 2:** Effect of microplastic quantity on DNA concentration and purity (*A*_260_/*A*_280_ ratios extracted from biofilm samples^*a*^

Treatment	DNA concentration (ng µL⁻¹)	*A*_260_/*A*_280_
Q30	13.9 ± 0.87^c^	0.18 ± 0.005^a^
Q50	16.7 ± 0.56^c^	0.19 ± 0.003^a^
Q100	22.3 ± 0.27^ab^	0.17 ± 0.007^a^
Q150	26.5 ± 3.93^a^	0.19 ± 0.012^a^

^*a*^ Treatments Q30-Q150 represent increasing microplastic loadings. Different letters (a > b > c) indicate statistically significant differences among treatments (*P* < 0.05; ANOVA followed by Tukey–Kramer post hoc test). Values are mean ± SD.

Finally, we validated the complete optimized protocol using field-collected MiPs from contrasting environmental conditions.

### Validation of the optimized method using two independent field-collected microplastics samples

To evaluate protocol robustness, MiPs collected from two environmentally distinct sites—surface water from Cau Den River, Hanoi (HN), and sediment from a clam farm in Nam Dinh (ND) were assessed. These sites represent contrasting ecological contexts—surface water MiPs develop biofilms under aerobic, high-light conditions, favoring planktonic colonizers, while sediment-associated MiPs experience reduced oxygen, greater organic matter content, and harbor benthic bacterial communities that produce highly adherent extracellular polymeric substances (EPS) conferring greater structural integrity to biofilms, as observed under SEM (Fig. S3).

Despite the MiPs originating from different environmental sites, the extraction efficiency remained consistent across samples (Table S2). Suspended biomass, quantified as OD_600_, was comparable between HN (0.032 ± 0.014) and ND (0.032 ± 0.086), indicating similar amounts of biofilm material released into the extraction suspension. Likewise, residual biofilm retained on MiP surfaces—measured as OD_595_—showed no significant difference between HN (0.037 ± 0.018) and ND (0.037 ± 0.007). For both metrics, t-tests yielded (independent t-test: *t* = 0, *P* > 0.05), confirming that site-specific differences in MiP origin did not affect overall extraction performance.

Viable cell recovery also remained robust across sites, with HN yielding 24,800 ± 8,900 CFU MiP⁻¹ and ND yielding 22,100 ± 6,700 CFU MiP⁻¹ (independent t-test: *t* = 0.331, *P* > 0.05), demonstrating that the protocol’s exceptional efficiency (>2,000-fold improvement over conventional methods) is maintained regardless of environmental origin. Similarly, DNA yields were comparable (HN: 24.1 ± 4.2 ng µL⁻¹; ND: 23.8 ± 3.8 ng µL⁻¹; independent t-test: *t* = 0.092, *P* > 0.05), supporting the use of this protocol across diverse aquatic matrices. This consistency is noteworthy given that, as reported in the literature (22, 31), extraction methods often show higher variability when applied to environmentally weathered compared to laboratory-incubated MiPs, a distinction that could not be directly compared within our own data set, as no laboratory-incubated comparator was included in this study (22, 31), confirming the protocol’s suitability for standardized environmental monitoring programs.

In summary, the optimized improved-efficiency protocol combining PBS + 0.1% Tween 80, 10-min ultrasonication (40 kHz), vortexing with glass beads, and a sequential detachment-disaggregation workflow achieved viable cell recovery of 28,020 ± 11,034 CFU MiP⁻¹, representing a 2,950-fold improvement over conventional PBS extraction. The protocol maintained consistent performance across environmental samples (surface water and sediment). DNA yields were sufficient for targeted PCR-based pathogen detection; however, the low *A*₂₆₀/*A*₂₈₀ ratios (0.17–0.19) indicate that additional inhibitor-removal purification is strictly required prior to NGS or quantitative applications. This confirms the protocol’s dual functionality for both culture-dependent analyses and targeted molecular pathogen detection.

## DISCUSSION

### Surfactant-enhanced biofilm extraction

The use of PBS + 0.1% Tween 80 in significantly improving biofilm extraction reflects the critical role of non-ionic surfactants in disrupting biofilm adhesion. Tween 80, an amphiphilic polysorbate, reduces interfacial tension and solubilizes hydrophobic EPS components, facilitating detachment from MiP surfaces. While both Tween 20 and Tween 80 enhanced extraction, Tween 80’s longer oleic acid chain (C18:1) provides greater lipophilicity, potentially able to penetrate further into the EPS matrix of the biofilms and resulting in greater cell disaggregation. Our results here corroborate those of previous studies demonstrating Tween 80’s efficacy in disrupting *P. aeruginosa* and *E. coli* biofilms and enhancing bacterial recovery from environmental plastics (28, 32). This is the first systematic evaluation showing Tween 80 is more effective than Tween 20 for microplastic biofilms.

The two-step extraction protocol was critical for achieving complete release of biofilm-embedded bacteria. In particular, the sequential vortexing–sonication treatment in Tween 80 buffer (SV4) increased viable cell recovery by 208-fold compared with the initial extraction step alone (SF4). This substantial enhancement likely reflects the effective disaggregation of biofilm fragments generated during Step 1, allowing viable cells trapped within the exopolymeric matrix to be fully liberated. Similar to findings from classical biofilm studies, the combination of mechanical disruption and surfactant-mediated disaggregation is necessary to overcome the strong adhesive properties and structural integrity of EPS, which can tightly entrap microbial cells (33).

Effective disaggregation is particularly critical for accurately characterizing biofilm-associated microbial communities. Bacteria residing deep within biofilms—including pathogenic species and antibiotic-resistant populations—are often embedded in dense EPS layers and form multicellular aggregates that resist detachment (34). Without surfactant-mediated disruption, a significant proportion of these cells would remain unrecovered, resulting in underestimation of microbial abundance, biased community profiles, and potentially misleading conclusions regarding pathogen or AMR gene prevalence. Such inaccuracies represent a major bottleneck in plastisphere research and can obscure risk assessments related to MiP-associated microbes and their implications for human and animal health, aquatic ecosystems, and industries such as aquaculture (35, 36).

### Sonication optimization and viability preservation

Ultrasonication is widely used for biofilm detachment because acoustic cavitation generates strong shear forces that disrupt extracellular polymeric substances (EPS) and release embedded cells (37). Low-frequency sonication (<50 kHz) produces alternating compression–rarefaction cycles that induce bubble formation and collapse, generating localized hydrodynamic forces capable of disrupting biofilm structure and weakening biofilm–substrate adhesion (38). In this study, sonication at 40 kHz for 10 min empirically maximized viable cell recovery within the tested duration range, yielding 15.2-fold and 9.1-fold higher CFU recovery compared with passive extraction (C1) and vortexing alone (C2), respectively.

Although optimization was evaluated using culturability as the primary outcome, this approach does not imply the absence of non-culturable or viable-but-non-culturable (VBNC) cells. CFU counts were intentionally applied as a viability-sensitive metric to assess physical detachment efficiency. Non-culturable community members remain associated with the extracted biofilm matrix and can be recovered through downstream molecular analyses, such as DNA-based workflows. Accordingly, the optimization strategy aimed to maximize detachment efficiency while preserving viability where possible, without excluding the broader microbial community.

Unlike high-frequency or prolonged sonication (>100 kHz, >20 min) that reduces bacterial survival (29), our moderate conditions (40 kHz, 10 min) combined with Tween 80 likely minimized cavitational stress and membrane damage. Tween 80 may stabilize cell membranes by forming protective interfacial layers. The 10-min exposure achieves optimal balance—sufficient to disrupt EPS and release cells while preserving viability, consistent with previous reports on biofilm recovery from biomedical materials (39).

Vortexing with glass beads provided complementary mechanical disruption through turbulent flow and surface abrasion. When combined, sonication weakened the EPS matrix and biofilm attachment, while vortexing enhanced physical removal. This synergistic approach outperformed individual treatments, in agreement with Stevenson (31). Importantly, our study extends these findings to environmentally weathered MiPs, demonstrating that optimized combinations of surfactants, sonication, and mechanical agitation are required to overcome the strong adhesion of aged, multilayered biofilms characteristic of environmental MiPs (40).

### Advancing beyond existing methodologies: systematic optimization vs. method comparison

Our work builds upon but significantly advances two key prior studies. Stevenson compared four extraction methods and identified sonication–vortexing as superior for culturability (31). However, their study was fundamentally comparative in design—testing fixed parameters across different approaches—rather than systematically optimizing individual variables to maximize performance. Critically, their study focused on pristine polypropylene pellets under controlled laboratory conditions, which lack the surface weathering, organic matter coating, and multilayered biofilm complexity characteristic of environmental MiPs (4, 20).

Our multi-factorial optimization revealed that synergistic interactions between surfactant type, mechanical disruption intensity, and sequential processing determine overall performance. For example, while Stevenson et al. used sonication, they did not systematically evaluate duration effects or quantify viability preservation. Our finding that 40 kHz sonication for 10 min achieves optimal EPS disruption without cell damage—yielding 15.2-fold higher CFU recovery than passive extraction—represents a critical refinement. Similarly, our demonstration that Tween 80 outperforms Tween 20 for microplastic biofilms addresses a gap left by previous studies that mentioned surfactants without comparative evaluation.

Debeljak et al. provided valuable insights into DNA extraction efficiency from ocean MiPs, observing that recovery scaled with particle surface area (22). However, their study focused on comparing commercial DNA extraction kits—representing downstream chemical lysis optimization—while employing relatively simple upstream physical detachment (shaking in PBS). They noted substantial variability in DNA yields but did not investigate whether this stemmed from incomplete biofilm detachment, cell lysis during extraction, or matrix interference. Our work addresses this gap by systematically optimizing the upstream biofilm detachment process before DNA extraction, thereby improving both DNA yield (26.5 ± 3.93 ng µL⁻¹ from 150 particles) and, critically, viable cell recovery for culture-based analyses.

Neither previous study achieved dual optimization for both cultivation and molecular approaches—a requirement for comprehensive plastisphere characterization under One Health frameworks where both pathogen isolation (for antimicrobial susceptibility testing, virulence profiling) and community-level molecular analyses (for ARG detection, taxonomic profiling) are essential (13, 18). Our protocol simultaneously delivers high viable cell counts (28,020 ± 11,034 CFU MiP⁻¹) and sufficient DNA quality for PCR-based pathogen detection, addressing a critical unmet need in the field.

Perhaps most significantly, existing studies provided limited quantitative benchmarks for extraction performance, making it difficult for other researchers to assess protocol efficiency or troubleshoot suboptimal results. By reporting comprehensive metrics—including viable cell recovery rates, DNA yields, extraction efficiency (OD_600_, residual OD_595_), and coefficients of variation—we establish quantitative standards that enable meaningful cross-study comparisons and protocol validation. This standardization is essential for advancing plastisphere research from exploratory studies toward reproducible, quantitative science suitable for regulatory risk assessment.

### Microplastic quantity and DNA quality considerations

Determining minimum MiP quantities is crucial for field-based plastisphere research, where manual isolation from environmental matrices is highly labor-intensive. Our results show that 100–150 MiP particles provide an optimal balance between biomass recovery and practicality, yielding >15,000 CFU MiP^−1^ and >20 ng µL⁻¹ DNA, sufficient for microbial characterization via culture-based methods, qPCR, and 16S rRNA sequencing. Smaller quantities (30–50 MiPs) remain feasible but yield higher variability and reduced sensitivity for low-abundance taxa or genes, which can limit pathogen or antibiotic resistance detection.

The positive correlation between MiP particle number and DNA yield supports findings by Debeljak et al., who observed DNA recovery scaling with particle surface area (22). Larger MiPs often host thicker biofilms, whereas highly weathered smaller MiPs—with rough microcracked surfaces—may trap cells more firmly, reducing detachment efficiency. Such size-dependent variability underscores the need for further studies, especially on nanoplastics (<1 µm), which are increasingly recognized as microbial vectors (41).

Consistently low DNA purity ratios (*A*₂₆₀/*A*₂₈₀ = 0.17–0.19) reflect the complexity of environmental biofilms, which contain massive amounts of proteins, humic substances, and EPS that co-extract with nucleic acids (33, 42). EPS components, particularly exopolysaccharides, strongly interfere with spectrophotometric quantification and downstream enzymatic reactions. Despite this severe matrix contamination, the isolated raw DNA empirically supported successful targeted PCR amplification of specific short-amplicon marker genes (*Aeromonas* spp., *S. enterica*). However, we must strongly caution that these low-purity extracts are fundamentally incompatible with highly efficient molecular workflows. Rigorous inhibitor-removal chemistries and stringent purification using commercial column-based kits are strictly required before these extracts can be utilized for sensitive next-generation sequencing (NGS) or quantitative PCR (qPCR) applications (43, 44).

### Environmental validation demonstrates protocol robustness

The consistent extraction efficiency observed between surface water (Cau Den River, Hanoi) and sediment (clam farm, Nam Dinh) MiPs validates the protocol’s robustness across contrasting environmental conditions. These two sites represent fundamentally different ecological contexts: surface water MiPs develop biofilms under aerobic, high-light conditions with lower organic matter content, whereas sediment-associated MiPs experience reduced oxygen availability, elevated organic matter, and distinct microbial communities that produce more recalcitrant extracellular polymeric substances (EPS).

SEM imaging revealed that sediment-derived biofilms exhibited greater structural complexity and denser EPS matrices compared to surface water samples (Fig. S3), consistent with observations that anaerobic conditions promote enhanced EPS production as a protective response (45). Despite these biofilm architectural differences, extraction performance remained statistically indistinguishable between sites (OD_600_: HN 0.032 ± 0.014 vs. ND 0.032 ± 0.086; independent t-test: t = 0, *P* > 0.05; residual OD_595_: HN 0.037 ± 0.018 vs. ND 0.037 ± 0.007; independent t-test: t = 0, *P* > 0.05). This consistency demonstrates that the optimized combination of Tween 80 surfactant and mechanical disruption (10-min sonication + vortexing with glass beads) effectively overcomes the enhanced adhesive properties of sediment biofilms without requiring site-specific protocol adjustments.

Environmentally weathered MiPs host highly complex, multi-layered biofilms and organic coatings that are reported to exhibit significantly higher resistance to extraction and greater analytical variability compared to the relatively simple biofilms formed on laboratory-incubated plastics (46, 47). The protocol’s performance consistency across environmental gradients is particularly significant given that plastisphere biofilm architecture varies substantially with environmental parameters, including oxygen availability, nutrient status, light exposure, and sediment organic carbon content (45). Previous extraction methods, particularly those developed using laboratory-incubated virgin plastics, often fail when applied to environmentally weathered MiPs that host multilayered, compositionally complex biofilms reinforced by adsorbed natural organic matter, metal oxides, and microbially mediated surface degradation (20, 40). Our validation using field-collected, naturally colonized MiPs confirms the protocol is suitable for real-world environmental monitoring applications in which MiP surface properties and biofilm characteristics vary widely.

This robustness has direct implications for large-scale environmental surveillance programs. Standardized methods that perform reliably across diverse matrices—freshwater vs. marine, planktonic vs. benthic, oligotrophic vs. eutrophic systems—are essential for generating comparable data sets that support meta-analyses and risk assessment frameworks. The demonstrated consistency across aerobic-anaerobic gradients suggests the protocol will likely perform well in other aquatic systems, including wastewater treatment plants, estuarine environments, and marine sediments, though systematic validation in these additional matrices would further strengthen its applicability.

### Biofilm biomass vs. viable cell recovery: understanding the disaggregation bottleneck

A key outcome of the optimization experiments was the pronounced mismatch between apparent biofilm removal and viable cell recovery during single-cycle extraction. Specifically, in Step 1, surfactant-containing solutions (SF3 and SF4) achieved the highest levels of biofilm detachment, as indicated by elevated OD_600_ values (0.042–0.046), yet resulted in surprisingly low recoverable cell numbers (10–26 CFU MiP⁻¹). This apparent paradox can be explained by the structural organization of biofilms and the mechanisms governing their disruption.

Optical density measurements capture total suspended particulate matter, encompassing intact biofilm fragments, extracellular polymeric substance (EPS) aggregates, cellular debris, and free cells. Upon mechanical or chemical perturbation, biofilms are typically released from surfaces as multicellular aggregates rather than as fully dispersed single cells, a behavior well documented in biofilm systems (33, 34). Consequently, although these aggregates are effectively detached from MiP surfaces—leading to high OD_600_ readings—most viable cells remain embedded within EPS matrices. When such material is plated directly, each aggregate generally yields a single colony regardless of the number of viable cells it contains, resulting in a substantial underestimation of true bacterial abundance.

The 208-fold increase in CFU recovery observed following Step 2 disaggregation (SV4: sonication and vortexing in PBS supplemented with 0.1% Tween 80) directly overcomes this limitation. The amphiphilic nature of Tween 80 disrupts hydrophobic interactions within the EPS, while combined mechanical agitation fragments multicellular aggregates, thereby releasing individual cells capable of forming discrete colonies. Importantly, this disaggregation step is mechanistically distinct from initial biofilm detachment and represents a critical, yet often overlooked, bottleneck in biofilm extraction workflows.

These findings have important methodological implications for plastisphere research. Studies relying on single-step extraction protocols—even those incorporating sonication or surfactants—are likely to recover only a small fraction of the viable microbial community. Such underestimation may systematically bias community profiles toward loosely attached or easily dispersed taxa, while underrepresenting deeply embedded populations that may include opportunistic pathogens, antibiotic-resistant bacteria, or highly specialized biofilm formers (48). Notably, taxa such as *Pseudomonas aeruginosa* and *Vibrio* spp. are known to produce dense, EPS-rich biofilms that are particularly resistant to mechanical disruption (49); without effective post-detachment disaggregation, their association with MiPs would be markedly underestimated.

Taken together, the sequential two-cycle extraction-disaggregation framework constitutes a conceptual shift rather than a marginal technical refinement. Step 1 primarily optimizes physical detachment of biofilms from MiP surfaces, whereas Step 2 is essential for maximizing the release of individual viable cells from detached biofilm matrices. This dual optimization decouples detachment from recovery and is critical for generating accurate, quantitative assessments of plastisphere-associated microbiota, thereby advancing current methodological standards in the field.

### Implications, limitations, and future perspectives

This protocol requires only basic equipment (bath sonicator, vortex mixer, and standard microbiology tools) and modest reagent costs, making it accessible for resource-limited laboratories and large-scale monitoring. The two-step approach, while requiring additional time, significantly improves sensitivity and reproducibility.

While the optimized protocol demonstrated strong performance, several limitations should be acknowledged. First, the manual, particle-by-particle isolation workflow inherently limits the absolute throughput of the method. Second, broader evaluation across contrasting systems—marine vs. freshwater, tropical vs. temperate—would strengthen its general applicability, as marine MiPs host distinct biofilm assemblages shaped by salinity and nutrient regimes (50). Third, a notable limitation is the absence of non-plastic control particles (e.g., sediment grains or natural organic debris), which are essential to distinguish plastic-specific microbial risks from general environmental background communities. Finally, while using heterogeneous environmental mixtures accurately reflects field conditions, the specific polymer composition of these mixtures was not identified (e.g., via µFTIR). Future studies should pair this extraction protocol with rigorous polymer characterization.

Crucially, our protocol optimization relied heavily on CFU counts, thereby inherently reflecting only the culturable fraction of the plastisphere. Because sonication, surfactants, and handling stress can differentially affect diverse taxa, these extraction conditions may selectively bias community composition by favoring stress-tolerant or fast-growing organisms while potentially damaging sensitive species or disrupting extracellular DNA pools. Furthermore, in alignment with our One Health framework, this study explicitly prioritized the recovery of ubiquitous Gram-negative aquatic pathogens (*E. coli*, *P. aeruginosa*, *Aeromonas* spp., and *Salmonella* spp.). Consequently, while the protocol is highly effective for these critical waterborne indicators, its efficacy in extracting and preserving intact Gram-positive bacteria (with thick peptidoglycan layers) or acid-fast bacteria remains unverified. More broadly, this method focused on bacterial recovery; its applicability to other microbial groups (archaea, fungi, protozoa, and viruses) remains untested but represents a logical extension given the multi-kingdom nature of plastisphere ecosystems (51, 52).

The suboptimal DNA purity observed (*A*₂₆₀/*A*₂₈₀ ≈ 0.18) reflects EPS and protein contamination typical of environmental biofilms, but can be improved using commercial DNA purification kits with inhibitor-removal chemistries for high-resolution molecular analyses, including qPCR and metagenomics (53). Polymer type, particle size, and weathering degree may also influence extraction efficiency. Future studies should systematically evaluate these variables under controlled conditions, particularly for highly weathered MiPs where surface microcracks may trap cells and complicate detachment. Future research must also incorporate culture-independent total cell quantification (e.g., flow cytometry or confocal microscopy) to confirm whether the method evenly recovers the full microbial community across all major taxonomic groups.

Future research should prioritize automation and miniaturization of extraction workflows to enable higher throughput and single-particle microbiome characterization. Combining refined extraction with advanced multi-omics approaches—metagenomics, metaproteomics, metabolomics, and stable isotope probing—could yield mechanistic insights into plastisphere function and its role in biogeochemical cycling (54).

Taken together, the optimized sequential detachment-disaggregation protocol developed and validated in this study represents a significant methodological advance for environmental plastisphere research. By delivering substantially enhanced viable cell recovery (2,950-fold over conventional PBS extraction), consistent performance across contrasting aquatic matrices, and functional DNA suitable for targeted pathogen detection, this protocol overcomes the principal extraction bottlenecks that have limited the reliability of plastisphere microbiome data. Its practical accessibility—requiring only standard laboratory equipment and modest reagent costs—positions it as a broadly deployable tool for environmental monitoring programs. The explicit delineation of application tiers (direct PCR detection vs. NGS requiring additional purification) provides clear operational guidance for end users. Most importantly, by enabling accurate quantification of plastisphere-associated pathogens and antimicrobial resistance genes, this validated foundational protocol directly supports the generation of reliable, unbiased data required for One Health risk assessment frameworks governing microplastic-associated microbial hazards in aquatic ecosystems.

In conclusion, we have developed a highly efficient, sequential detachment-disaggregation protocol tailored for environmental microplastics. While this two-step workflow significantly mitigates the “disaggregation bottleneck,” we acknowledge its current limitations. The reliance on manual particle isolation limits automated high-efficiency scalability, yet it strategically preserves biofilm integrity during initial separation. Importantly, while using heterogeneous environmental mixtures accurately reflects field conditions, the lack of specific polymer identification is a limitation. Future validations across characterized polymer profiles are recommended before this protocol can be fully adopted as a validated, robust foundational tool for global environmental MiP biofilm surveillance.

## MATERIALS AND METHODS

### Solutions and materials used

Phosphate-buffered saline (10 mM, pH 7.2–7.4; hereafter referred to as 1× PBS) was prepared by mixing NaH₂PO₄ and Na₂HPO₄ (Merck, Germany) at a 2:8 ratio with 22.8 g L^−1^ of NaCl in distilled water (DI). Surfactants Tween 20 and Tween 80 (Biobasic, Canada) were prepared as 10% (vol/vol) stock solutions and diluted to 0.1% (vol/vol) with 1× PBS for use. Nutrient agar (NA) contained 10 g L^−1^ peptone, 5 g L^−1^ yeast extract, 5 g L^−1^ NaCl, and 15 g L^−1^ agar (Difco, USA). Crystal violet solution (0.1% wt/vol; Sigma-Aldrich, USA) and ethanol (70% and 95%; Merck, Germany) were used for staining, elution, and sterilization.

Sterile solid-glass beads (~3 mm diameter, autoclaved at 121°C for 20 min) (Merck, Germany), stainless steel sieves (80 µm mesh; Endecotts, UK), metal filter membranes (47 mm diameter, 80 µm pore size), and powder-free laboratory wipes (Kimtech Kimwipes, USA) were used throughout the study.

### Sediment sampling and handling

Surface sediment samples (0–10 cm in depth) were collected from the Day River (20°29′29″N, 105°53′34″E) in Ha Nam Province, Vietnam, during April 2025. To minimize contamination, sampling was conducted using ethanol-sterilized stainless-steel spoons. Immediately following collection, sediment samples were sealed in pre-cleaned aluminum foil bags (rinsed three times with 70% ethanol and air-dried) and maintained at 4°C during transport to the laboratory (within 6 h of collection).

Upon arrival, samples were processed under aseptic conditions. Visible plant debris, shells, and coarse particles (>2 mm) were manually removed using sterile forceps. The remaining sediment was thoroughly homogenized and transferred to sterile sealed containers and stored at 4°C for subsequent MiP isolation (within 7 days of collection).

### Microplastic isolation and characterization

Sediment samples (50 g wet weight) were suspended in 200 mL of 1× PBS and gently stirred for 5 min (200 rpm; VM-30, Daihan Scientific, Korea) to release MiPs. The suspension was filtered through a sterile 80 µm stainless-steel sieve using a vacuum system (Rocker 300, Taiwan; −50 kPa) and rinsed three times with 50 mL 1× PBS to recover retained particles.

Filters containing MiPs and residual matter were placed in sterile Petri dishes and examined under a stereomicroscope (Euromex SB1903, 10–40×). MiPs were visually identified based on morphological criteria (55) to be isolated using sterile forceps (Dumont #5, Switzerland) and transferred into 1.5 mL tubes with 1 mL PBS. Samples were then stored at 4°C overnight prior to biofilm extraction.

Crucially, to maintain ecological validity and represent the objective heterogeneity of the bulk environmental matrix, the isolated MiPs were deliberately pooled into a single heterogeneous “master mixture” without any prior sorting by specific polymer types (e.g., polyethylene vs. polypropylene), exact sizes, or precise shapes. All subsequent biological replicates were generated by randomly drawing independent aliquots (e.g., 50 particles) from this master pool, thereby ensuring true statistical independence and avoiding pseudo-replication across treatments.

To remove loosely attached material from the isolated MiPs (organic matter, sediment particles, and microorganisms), MiPs were subjected to a standardized cleaning protocol. Individual MiPs were transferred from storage tubes to sterile Petri dishes and rinsed three times with 1 mL of 1× PBS using sterile pipettes. After each rinse, the MiP surface was inspected under the stereomicroscope (40× magnification) to confirm the absence of visible contaminants. Washed MiPs were then dried by gently pressing them between powder-free laboratory wipes (Kimtech Kimwipes) and allowed to air-dry in a sterile laminar flow hood for 10 min before biofilm extraction procedures.

### Optimization of the biofilm extraction workflow

**Fig 2:**
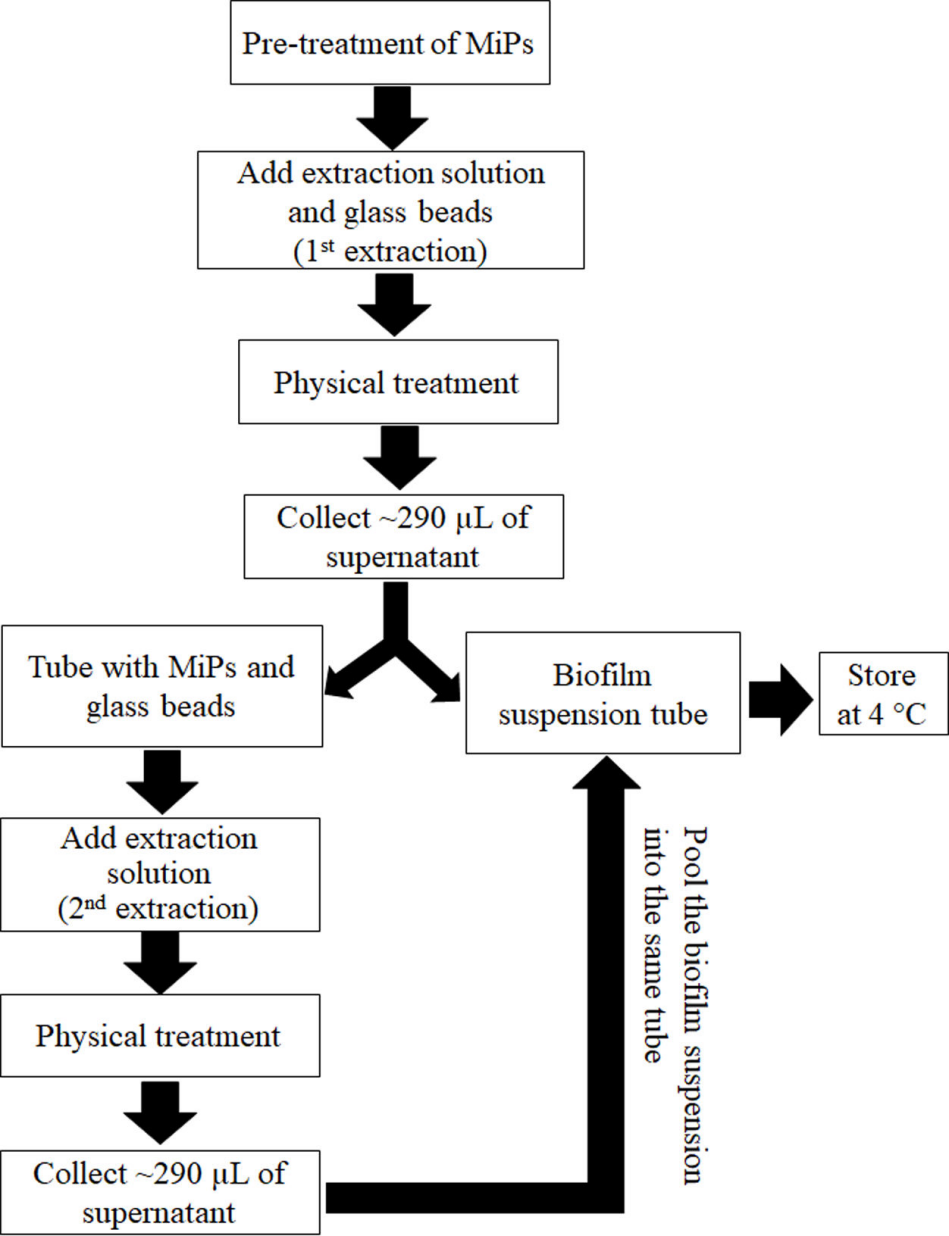
Workflow for extracting biofilms from microplastic (MiP) surfaces. The optimized protocol uses phosphate-buffered saline (PBS) supplemented with 0.1% Tween 80, followed by sequential physical treatments consisting of ultrasonication (40 kHz, 10 min) and vortexing (3,000 rpm, 2× 30 s).

The biofilm extraction protocol consisted of sequential mechanical and chemical disruption steps designed to maximize biofilm recovery while maintaining cell viability (Fig. 2). Briefly, pre-cleaned MiPs were placed in microcentrifuge tubes containing extraction solution (1× PBS), glass beads, and subjected to ultrasonication followed by vortexing. This two-step extraction process was repeated, and the resulting suspensions were pooled for downstream microbial analysis. Residual biofilm remaining on MiP surfaces was quantified using crystal violet staining to assess extraction efficiency. The biofilm extraction protocol was systematically optimized to maximize extraction efficiency while maintaining bacterial viability. Three key parameters were evaluated: (i) extraction solution composition, (ii) physical extraction parameters, and (iii) quantity of MiPs used.

#### Effect of extraction solution and mechanical processing

To optimize biofilm extraction while preserving microbial viability, four test solutions were independently evaluated: (i) 0.85% (wt/vol) NaCl; (ii) 1× PBS; (iii) 1× PBS + 0.1% (vol/vol) Tween 20; and (iv) 1× PBS + 0.1% (vol/vol) Tween 80.

To achieve optimized viable cell recovery from MiP biofilms, solution selection was based on established biofilm disruption mechanisms: NaCl provides isotonic baseline conditions; PBS adds buffering capacity for pH stability; non-ionic surfactants (Tween 20 and Tween 80) enhance detachment by reducing surface tension and disrupting hydrophobic EPS-MiP interactions with none or minimal lysing of cells.

##### Step 1: Chemical-mechanical detachment–Solution composition screening (Treatments FS1–FS4)

Environmental MiPs (50 particles per replicate, *n* = 3) were transferred to 1.5 mL microcentrifuge tubes containing 300 µL of test solution and five sterile glass beads (3 mm diameter). Biofilm extraction was performed using sequential mechanical disruption: ultrasonication (40 kHz, 100 W, 10 min; Au-220, Agro Lab, Italy) followed by vortexing (3,000 rpm, 1 min; VM-30, Daihan Scientific, Korea).

Following the first extraction, 290 µL of each suspension (hereafter defined as biofilm suspension) was carefully transferred to the fresh 1.5 mL microcentrifuge tube. The retained MiPs in the original tube for each treatment underwent a second identical extraction with 300 µL of fresh test solution. The two sequential extracts were pooled to yield combined biofilm suspensions for treatments FS1–FS4, corresponding to the four test solutions (respectively, NaCl; 1× PBS; 1× PBS + Tween 20; and 1× PBS + Tween 80).

A 100 µL aliquot from each pooled biofilm suspension was diluted 1:10 (vol/vol) in 1× PBS to determine extraction efficiency. The remaining volume for each of the biofilm suspensions was stored at 4°C for subsequent processing in the disaggregation step (described below; Table 1).

##### Step 2: Chemical disaggregation optimization (Treatments SV1–SV4*)*

Because bacteria within MiP-associated biofilms are tightly embedded in exopolymeric matrices, an additional disaggregation step was required after biofilm extraction to effectively release these cells for downstream analyses (e.g., bacterial quantification, pathogen detection, and ARG profiling). To optimize this release, the stored suspensions obtained from the first extraction step were subjected to a two-factor experiment evaluating disaggregation buffer (1× PBS vs*.* 1× PBS + 0.1% Tween 80) and the sequence of mechanical disruption (vortexing → sonication vs. sonication → vortexing). Suspensions were diluted 1:10 (vol/vol) into the test solutions and processed using either (i) double vortexing (3,000 rpm, 30 s × 2) followed by a single 10-min sonication at 40 kHz or (ii) the reverse order of these steps. These combinations were designated as treatments SV1–SV4 (Table 1). Tween 80 was introduced at this stage to promote disaggregation of bacterial clusters within the biofilm matrix, thereby improving the release and recovery of viable cells.

### Effect of sonication time on extraction efficiency

Using the optimal extraction solution (ES) from “Effect of extraction solution and mechanical processing,” above, the following five treatments were evaluated to determine the influence of sonication time on biofilm recovery (Table 3):

- **C1**: No mechanical disruption (passive release control)
- **C2**: Vortexing only (2× 30 s, no sonication)
- **T1**: 3-min sonication + two rounds of 30 s vortexing
- **T2**: 5-min sonication + two rounds of 30 s vortexing
- **T3**: 10-min sonication + two rounds of 30 s vortexing

**TABLE 3:** Experimental treatments for evaluating sonication duration effects on biofilm DNA extraction efficiency^*a*^

Treatment	Sonication duration	Vortexing	Extraction solution	Purpose
C1	None	None	ES (solution optimized)	Baseline: passive release
C2	None	2× 30 s	ES (solution optimized)	Vortex-only control
T1	3 min	2× 30 s	ES (solution optimized)	Low sonication exposure
T2	5 min	2× 30 s	ES (solution optimized)	Moderate sonication
T3	10 min	2× 30 s	ES (solution optimized)	Extended sonication

^*a*^ All treatments followed the two-cycle extraction and disaggregation protocol. Controls include no treatment (C1) and vortex-only (C2). Sonication durations of 3 min (T1), 5 min (T2), and 10 min (T3) were tested, each followed by standardized vortexing (2× 30 s). ES = extraction solution optimized as described in “Effect of extraction solution and mechanical processing”.

MiPs collected from the field (50 particles per replicate, *n* = 3) underwent a two-cycle extraction and disaggregation with 300 µL ES each. Biofilm suspensions were pooled for analysis. Sonication (40 kHz, 100 W) and vortexing (3,000 rpm) parameters were kept constant. While a deliberately lethal control (e.g., prolonged continuous sonication > 30 min) was not utilized to definitively quantify absolute cell lysis, the 10-min threshold was empirically selected as it maximized the recovery of viable cells relative to all other treatments without observable declines in CFU counts.

### Effect of microplastic quantity on extraction efficiency and reproducibility

To determine the minimum number of MiP particles that will yield efficient and reproducible biofilm extractions for downstream microbial analyses, four MiP quantities were tested: 30, 50, 100, and 150 particles per replicate (*n* = 6), respectively, Q30, Q50, Q100, and Q150. All extractions were performed using the optimized protocol identified in “Effect of extraction solution and mechanical processing” and “Effect of sonication time on extraction efficiency,” above.

### Evaluation of biofilm extraction efficiency

#### Viable cell enumeration

Viable bacterial number in biofilm suspensions was quantified by serial decimal dilution and spread plating on nutrient agar (NA). Plates were incubated at 37°C and colonies enumerated after 24–48 h. To monitor potential contamination and ensure that detected colonies originated solely from the extracted biofilms, a negative control was included in all assays. This control consisted of sterile PET MiPs processed using the same workflow as the test samples but without any biofilm extract added. Colony counts were reported as colony-forming units per MiP particle (CFU MiP^−1^), following the approach of Rajcoomar et al. (45, 50).

#### Residual biofilm quantification by crystal violet staining

Residual biofilm on extracted MiP surfaces was quantified using a modified crystal violet assay (48, 49). For this, following extraction, MiPs were stained with 500 µL of 0.1% (wt/vol) crystal violet for 45 min in the dark with gentle shaking (50 rpm). The particles were then washed three times with 1 mL PBS to remove unbound dye, air-dried for 45 min, and any MiP-bound dye was eluted with 1 mL of 95% ethanol (10 min in the dark) with intermittent vortexing. The resultant solutions (200 µL aliquots in Microplate Reader; Azure Ao, China) were analyzed by spectrophotometry at 595 nm. Values were normalized to MiP particle number (OD_595_/MiP); lower absorbance indicates higher biofilm removal efficiency. The crystal violet staining method was also applied to virgin PET MiPs (negative control) and pre-extracted environmental MiPs (positive control), and measured in parallel.

#### Assessment of total DNA extraction efficiency

Following the evaluation of the biofilm extraction procedure (see “Viable cell enumeration,” above), the remaining biofilm suspension was processed for total genomic DNA extraction using the DNeasy PowerSoil Pro Kit (Qiagen, Germany) with the following modifications. Biofilm suspensions were transferred into PowerBead Pro Tubes and centrifuged at 16,000 × *g* for 15 min at 4°C (Model Z36HK, HERMLE, Germany). After discarding the supernatant, 600 µL of Solution CD1 was added, and samples were incubated at 37°C for 30 min, followed by vortexing at 3000 rpm for 10 min (VM-30, Daihan Scientific, Korea). Pellets were resuspended in 200 µL sterile 1× PBS and transferred to PowerBead Pro Tubes.

Following cell lysis, the remaining steps of the DNA purification kit were performed according to the manufacturer’s instructions, from the step when Solution CD2 was added to precipitate humic acids and other PCR inhibitors, followed by centrifugation. The supernatant was transferred to a clean tube and mixed with Solution CD3 to bind DNA. The mixture was loaded onto an MB Spin Column, and DNA was bound to the silica membrane via centrifugation. The membrane was washed sequentially with Solutions EA and C5 to remove residual contaminants. Purified DNA was eluted by adding 50 µL of Solution C6 to the center of the membrane and centrifuging at 15,000 × *g* for 1 min.

DNA concentration and purity (*A*₂₆₀/*A*₂₈₀ ratio) were measured using a NanoDrop Lite spectrophotometer (Thermo Fisher Scientific, USA). Given the complexity of environmental plastisphere biofilm matrices, low *A*₂₆₀/*A*₂₈₀ ratios were expected due to co-extraction of EPS-derived proteins and humic substances; the extracted DNA was therefore evaluated functionally by its amplifiability in targeted PCR assays rather than by spectrophotometric purity criteria alone. The extracted DNA was subsequently utilized as a template to evaluate its amplifiability in targeted PCR assays.

PCR detection of *E. coli, P. aeruginosa, Aeromonas* spp*.,* and *S. enterica* was performed using GoTaq Green Master Mix (Promega, USA) in 25–50 µL reactions containing 2× Master Mix, gene-specific primers (0.4–1 µM; sequences in Table S1), and 1–5 µL template DNA. Cycling conditions: 95°C/5 min; 35 cycles of 95°C/30 s, annealing (temperature in Table S1)/30–60 s, 72°C/45 s; 72°C/10 min. Products were separated on 2% agarose gels, stained with GelRed, and visualized under UV light (ChemiDoc XRS+, Bio-Rad, USA).

### Validation of the optimized protocol with environmental microplastic samples

To evaluate the practical applicability of the optimized biofilm extraction protocol, validation experiments were conducted using environmental MiPs collected from surface waters at Cau Den, Hanoi (HN: 105°46′51″E, 20°58′24″N) and sediments from a clam farm in Nam Dinh (ND: 106°3′42″E, 20°16′45″N).

Water samples were pre-filtered through an 80 µm mesh, concentrated into 50 mL Falcon tubes, and transported to the laboratory. Samples were further filtered through an 80 µm stainless steel membrane and examined under a stereomicroscope. MiP particles were manually isolated using sterile tweezers and rinsed with 1× PBS. Sediment samples were processed as described in “Sediment sampling and handling” and “Microplastic isolation and characterization,” above

All recovered MiP particles were subjected to the optimized biofilm extraction protocol (see “Optimization of the biofilm extraction workflow,*”* above). This cycle was repeated twice, and suspensions were pooled. Extraction efficiency was assessed by quantifying residual biofilm via crystal violet staining (OD_595_) and measuring optical density (OD_600_) of the suspension (see “Evaluation of biofilm extraction efficiency,” above).

### Quality control and contamination prevention

To minimize contamination, all procedures from MiP isolation (see “Microplastic isolation and characterization,” above) through biofilm extraction (see “Optimization of the biofilm extraction workflow,” above) were conducted in a Class II biological safety cabinet (PURICUBE NEO 1200, Korea) with HEPA filtration and UV sterilization. Glassware, metal sieves, forceps, and glass beads were autoclaved (121°C, 20 min) and oven-dried (80°C, 2 h). PBS and dilution buffers were filter-sterilized (0.22 µm) and autoclaved. Work surfaces and equipment were disinfected with 70% ethanol before and after use. Personnel wore disposable PPE (laboratory coats, nitrile gloves, face masks), changed between batches, and maintained sterile zones using alcohol lamps during manual MiP handling.

For procedural controls, sterile virgin PET MiPs (70% ethanol, 30 min; dried at 70°C, 1 h) were processed in parallel to environmental samples, and samples of these were plated on NA to account for background contamination. In addition, negative extraction controls (extraction solution without MiPs) were included in DNA extraction batches to detect any contamination from any of the reagents used.

### Data analysis

All experiments were performed with a minimum of three biological replicates (*n* ≥ 3). Data are presented as mean ± standard error of the mean (SEM). Normality of distribution was assessed using the Shapiro–Wilk test. For data meeting normality assumptions, differences among treatment groups were evaluated using one-way analysis of variance (ANOVA) followed by Tukey’s Honest Significant Difference (HSD) post hoc test. For non-normally distributed data, the Kruskal–Wallis test followed by Dunn’s multiple comparison test was applied. Statistical significance was set at *P <* 0.05.

## Data Availability

Data will be made available on request.

## SUPPLEMENTAL MATERIAL

Supplemental material aem.00416-26-s0001.docx Tables S1 and S2; Fig. S1 to S3.

ASM does not own the copyrights to Supplemental Material that may be linked to, or accessed through, an article. The authors have granted ASM a non-exclusive, world-wide license to publish the Supplemental Material files. Please contact the corresponding author directly for reuse.
