# Supplementary material for: Developing an optimized method for biofilm extraction from microplastic surfaces for high-efficiency analysis of adherent bacterial communities
Source: Appl Environ Microbiol. 2026 Apr 29;92(5):e00416-26. doi: 10.1128/aem.00416-26 (PMC13188912; doi:10.1128/aem.00416-26)
Supplement: Supplemental material — Tables S1 and S2; Fig. S1 to S3. [file aem.00416-26-s0001.docx]

**Developing an Optimized Method for Biofilm Extraction from Microplastic Surfaces for High-** **Efficiency Analysis of Adherent Bacterial Communities**

Hieu Hoai Vo,^a,b,c^ Thao Thanh Le,^a,d^ Tu Van Nguyen,^a^ Jennifer Scott,^e^ Michel Kaiser,^f^ Tony Gutierrez,^e^ Huong Thi Thuy Ngo^a,d^#

*^a^Environmental Chemistry and Ecotoxicology Lab, Phenikaa School of Engineering, Phenikaa University, Ha Noi 12116, Viet Nam*

*^b^Yersin University of Da Lat, Lam Dong, Viet Nam*

*^c^National Institute of Hygiene and Epidemiology, Ha Noi, Viet Nam*

*^d^Faculty of Biotechnology, Chemistry and Environmental Engineering, Phenikaa School of Engineering, Phenikaa University, Ha Noi 12116, Viet Nam*

*^e^Institute of Mechanical, Process and Energy Engineering, School of Engineering and Physical Science, Heriot-Watt University, Edinburgh, UK*

*^f^The Lyell Centre, School of Energy, Geoscience, Infrastructure and Society, Heriot-Watt University, Edinburgh, UK*

# Address correspondence to Huong Ngo Thi Thuy, [huong.ngothithuy@phenikaa-uni.edu.vn](mailto:huong.ngothithuy@phenikaa-uni.edu.vn)

**Supplementary captions:**

**TABLE S1.** Primer sequences, amplicon sizes, and PCR cycling conditions for the target bacteria

**TABLE S2.** Validation of biofilm extraction method on field-collected microplastics: suspension turbidity (OD_600_), residual biofilm (OD_595_), and bacterial density (CFU per microplastic particle) from Vietnamese aquatic systems (HN = Hanoi urban waters; ND = Nam Dinh coastal waters) versus laboratory-optimized standard (Q100 = 100 MiP particles). CFU = colony forming units. Mean ± SD

**FIG S1.** Agarose gel electrophoresis results for PCR amplification of pathogen-specific genes from microplastic biofilm extracts. Gel (A) displays the 163 bp amplicons of *Salmonella* spp.; Gel (B) displays the 1236 bp amplicons of *Aeromonas* spp. Lanes: Ladder: DNA ladder; (+): Positive control; (-): Negative control; Q30, Q50, Q100, Q150: Biofilm DNA extracted from 30, 50, 100, and 150 microplastic particles, respectively. The suffixes .1, .2, and .3 denote three independent biological replicates.

**FIG S2.** Workflow for biofilm extraction from microplastic surfaces

**FIG S3.** Scanning electron microscopy (SEM) images illustrating the contrasting biofilm architectures on field-collected microplastics. (A) Microplastics from surface water (Cau Den, Hanoi - HN) showing a diverse but relatively thin microbial layer with visible bacterial cells and organic debris. (B) Microplastics from sediment (Clam farm, Nam Dinh - ND) displaying a highly complex, dense extracellular polymeric substance (EPS) matrix that deeply embeds microbial communities within surface microcracks. Scale bars represent 5 µm.

**Table S1.** Primer sequences, amplicon sizes, and PCR cycling conditions for the target bacteria

| **No.** | **Target** | **Sequencing** | **Amplicon size (bp)** | **PCR cycling program** | **Reference** |
| --- | --- | --- | --- | --- | --- |
| 1 | *Salmonella* spp. | SalF- 5’-TTA TTA GGA TCG CGC CAG GC-3’  SalR – 5’-AAA GAA TAA CCG TTG TTC AC-3’ | 163 | **Step 1:** 94ºC 1 min;  **Step 2: 36 cycles** of: 94ºC 1 min, 50ºC 1 min, 72 ºC 1 min;  **Step 3:** final 72ºC 5–10 min;  **Step 4:** hold 4ºC. | Espinoza-Medina et al. (2006)  *https://doi.org/10.4315/0362-028X-69.6.1422* |
| 2 | *Aeromonas* spp. | PF1 - 5′-TTTGGAACCCATTTCTCGTGTGGC-3′  PR1- 5′-TCGAAGTAGTCCGGGAAGGTCTTGG-3′ | 1236 | **Step 1:** 94ºC 2 min;  **Step 2:** **40 cycles** of: 92ºC 1 min, 50ºC 1 min, 72ºC 1 min;  **Step 3:** 72ºC 10 min;  **Step 4:** hold 4 ºC. | Cascón Soriano et al. (1997)  *https://doi.org/10.1111/j.1574-6968.1997.tb12727.x* |
| 3 | *Pseudomonas aeruginosa* | PASSF-5’-GGGGGATCTTCGGACCTCA-3’  PASSR-5’-TCCTTAGAGTGCCCACCCG-3’ | 956 | **Step 1:** 95ºC 2 min;  **Step 2:** **25 cycles** of: 94ºC 20 s, 58ºC 20 s, 72ºC 40 s;  **Step 3:** 72 ºC 1 min;  **Step 4:** hold 4ºC. | Spilker et al. (2004)  *https://doi.org/10.1128/jcm.42.5.2074-2079.2004* |
| 4 | *Escherichia coli* | cydAF*- 5’-*CGTATGGAGATGGTGAG-3’  cydAR – 5’- GTAGAACCAGAACGCAGT-3’ | 515 | **Step 1:** 95ºC 2 min;  **Step 2:** **35 cycles** of: 95ºC 30 s, **55ºC** 30 s, 72ºC 30 s;  **Step 3:** 72ºC 5 min;  **Step 4:** hold 4ºC. | Zimoń et al. (2024)  *https://doi.org/10.1128/spectrum.03773-23* |
|  |  | LacYF-5’- TTCCCACCGATGCGATT-3’  LacYR-5’- GTCACTGTATGTTATTGGCG-3’ | 192 |  |  |
|  |  | ydiVF-5’- CCATTTCTCCAGTGAAGAT-3’  ydiVR-5’- CCTAACACAAGGGGATAC-3’ | 330 |  |  |

**TABLE S2.** Validation of biofilm extraction method on field-collected microplastics: suspension turbidity (OD_600_), residual biofilm (OD_595_), and bacterial density (CFU per microplastic particle) from Vietnamese aquatic systems (HN = Hanoi urban waters; ND = Nam Dinh coastal waters) versus laboratory-optimized standard (Q100 = 100 MiP particles). CFU = colony forming units. Mean ± SD

| **Samples** | **Suspension OD₆₀₀** | **Residual biofilm OD₅₉₅** | **Bacterial density (CFU MiP^-1^)** |
| --- | --- | --- | --- |
| HN | 0.032±0.014 | 0.037±0.018 | 24,227±8,900 |
| ND | 0.032±0.086 | 0.037±0.007 | 22,100±6,700 |
| Optimization reference (Q100) | 0.028±0.009 | 0.041±0.001 | 20,340 ± 3,553 |

| 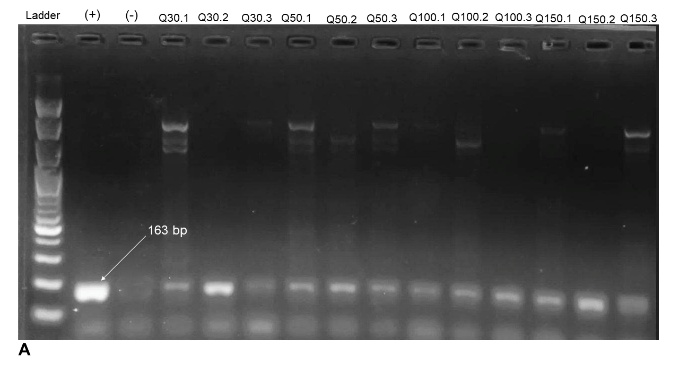 | 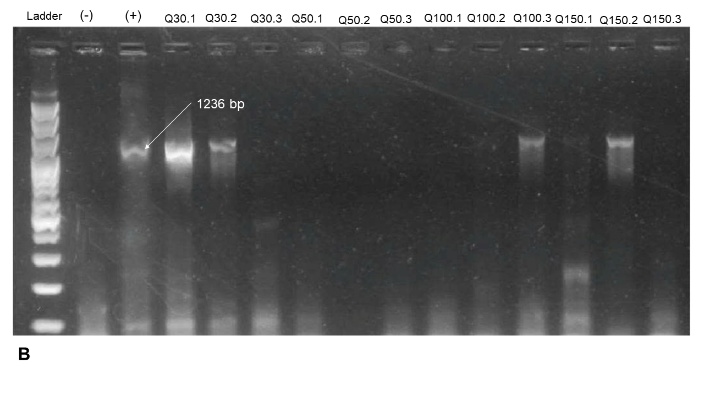 |
| --- | --- |

**Figure S1.** Agarose gel electrophoresis results for PCR amplification of pathogen-specific genes from microplastic biofilm extracts. **(A)** displays the 163 bp amplicons of *Salmonella* spp.; **(B)** displays the 1236 bp amplicons of *Aeromonas* sp. Lanes: Ladder: DNA ladder; (+): Positive control; (-): Negative control; Q30, Q50, Q100, Q150: Biofilm DNA extracted from 30, 50, 100, and 150 microplastic particles, respectively. The suffixes .1, .2, and .3 denote three independent biological replicates.


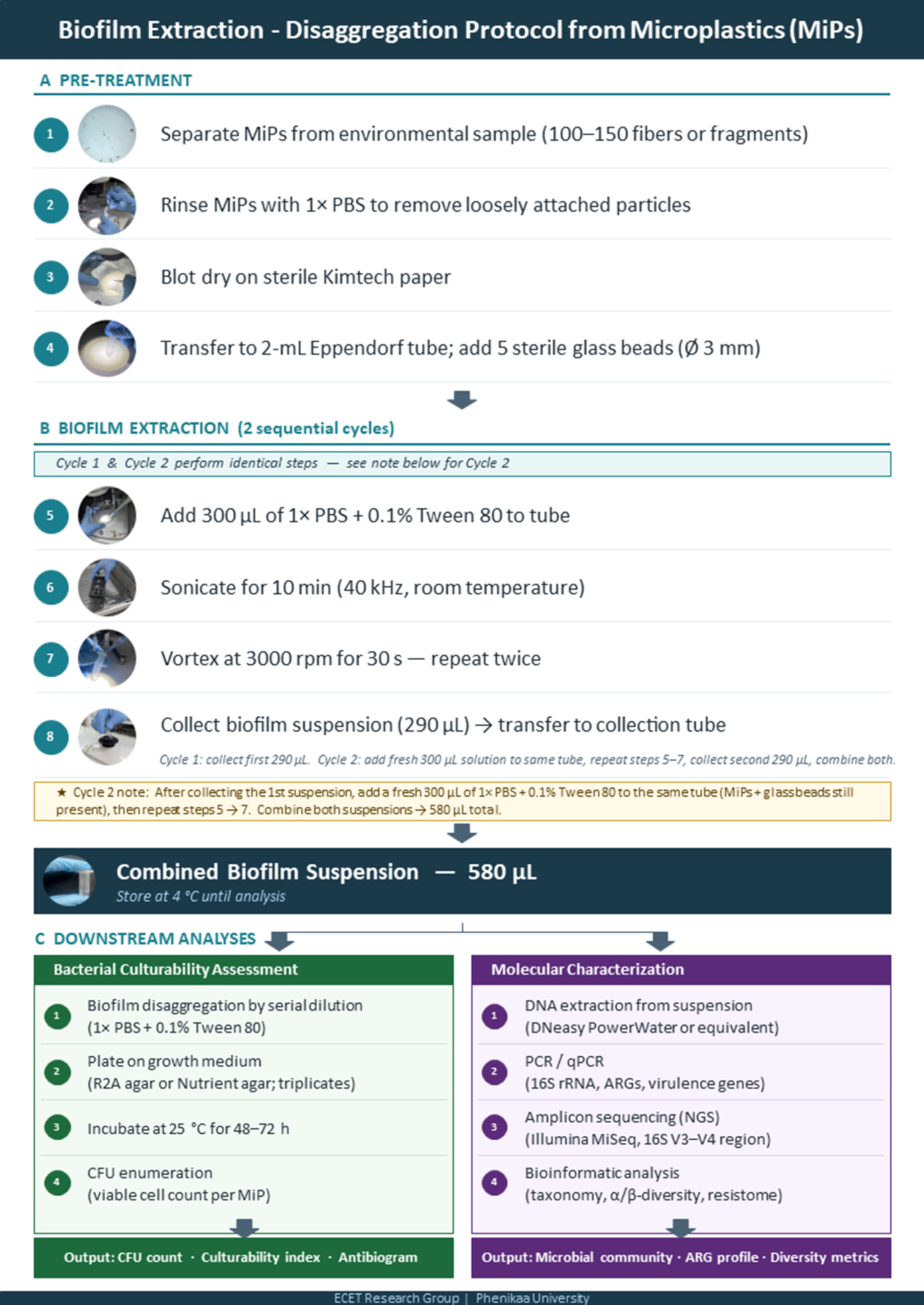


**Figure S2.** Schematic workflow illustrating the two-cycle sequential extraction and disaggregation of biofilm from microplastic surfaces. Figure generated with AI assistance and manually verified for scientific accuracy.

**
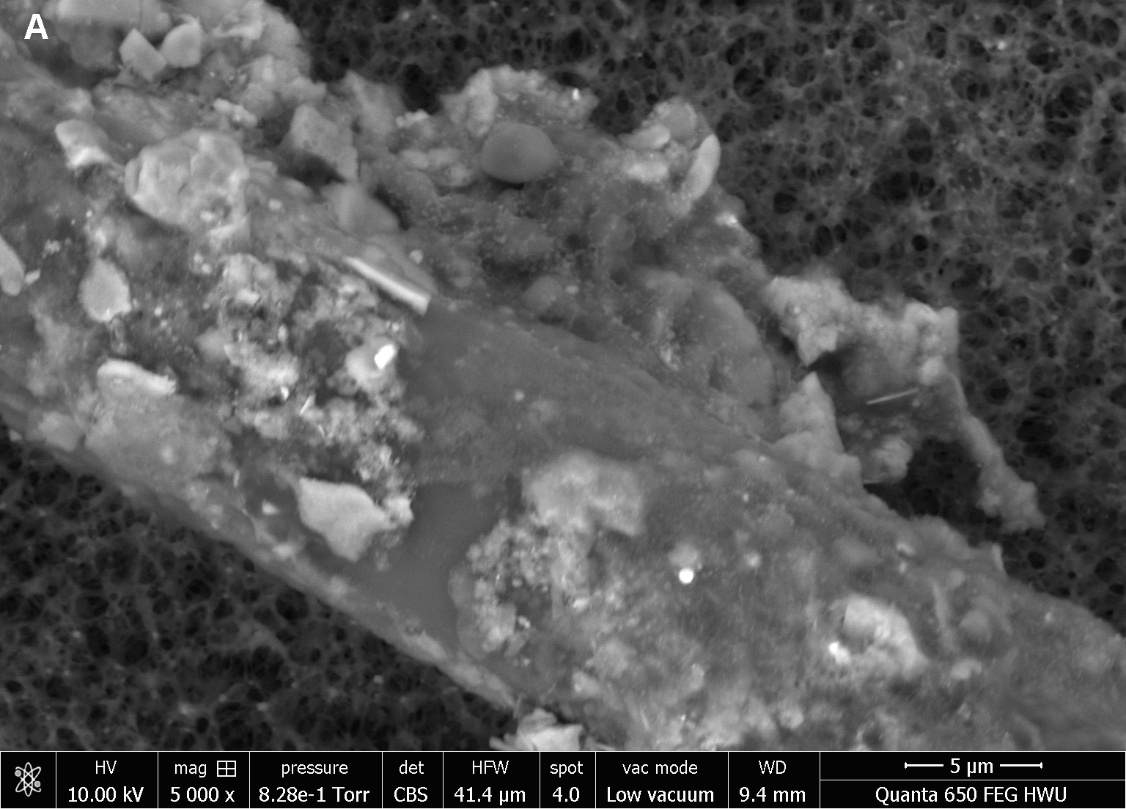
**

**
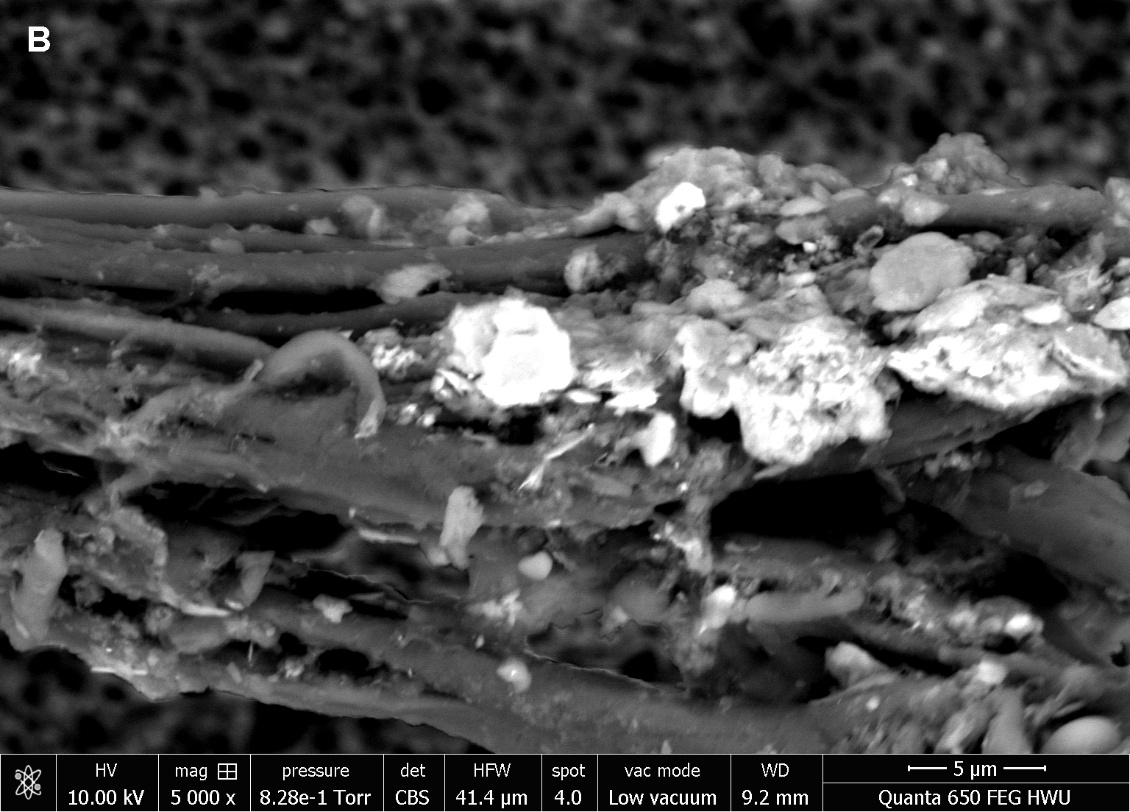
**

**Figure S3.** Scanning electron microscopy (SEM) images illustrating the contrasting biofilm architectures on field-collected microplastics. **(A)** Microplastics from surface water (Cau Den, Hanoi - HN) showing a diverse but relatively thin microbial layer with visible bacterial cells and organic debris. **(B)** Microplastics from sediment (Clam farm, Nam Dinh - ND) displaying a highly complex, dense extracellular polymeric substance (EPS) matrix that deeply embeds microbial communities within surface microcracks. Scale bars represent 5 µm.
